# Supplementary material for: Constraints on green renovation of building based on the theory of whole lifecycle and green development
Source: PLoS One. 2025 Jul 10;20(7):e0328148. doi: 10.1371/journal.pone.0328148 (PMC12244562; doi:10.1371/journal.pone.0328148)
Supplement: Attachment — Submitted filename: Response to reviewers.doc [file pone.0328148.s002.doc]

PONE-D-25-06830

**PLOS ONE**

We appreciate the opportunity to revise our manuscript titled " **Constraints on Green Renovation of Buildings Based on the Theory of Whole Lifecycle and Green Development** " and are grateful for the insightful comments provided by the reviewers. These comments are valuable and very professional for revising and modifying our paper, as well as the important guiding significance to our researches. In the resubmission, we have provided detailed responses to the reviewers' comments. Revised portion are marked in red in the revised manuscript. We have tried our best to make all the revisions clear, and we hope that the revised manuscript meets the requirements for publication. Here are the point-by point responses:

Reviewer #1: This paper proposes a system dynamics-based model for evaluating the comprehensive benefits of green building retrofits, analyzing the constraints and limitations faced during the retrofit process. The study, grounded in the theories of life cycle assessment and green development, simulates key indicators at various stages of green building retrofits, including pollution, greening, energy consumption, and resident satisfaction. It also emphasizes the significant role of engineering warranties and post-assessment phases in enhancing the greening level of buildings. The research holds certain value.

Reply: Thank you very much for your affirmation and evaluation of the manuscript.

1.Analysis diagrams such as Fig. 1, "Management organization system for GRB based on WLT," which organizes various roles and responsibilities involved in green building retrofit projects through conceptual analysis and transformation history of GRB, and proposes a management organization system for GRB based on WLT, lack clear explanation of their specific literature sources and basis.

Reply: Thank you very much for your valuable feedback on the manuscript. The literature source and basis for Figure 1 have been revised as follows.

The WLT has a central place in the GRB system, stressing the need of auditing and monitoring at every phase, from project design and development to construction and settlement. The thoroughness and real-time nature of GRB may be guaranteed by developing a system based on the whole lifespan. Through the conceptual analysis and transformation history of GRB, the various roles and responsibilities involved in the GRB project are sorted out, thereby constructing the management organization system. The GRB management organization system based on WLT is proposed, as shown in **Fig. 1 ^[19]^**.

19.Aly IA, Kurttisi A, Dogan KM. Resilient coordination of multi-agent systems in the presence of unknown heterogeneous actuation efficiency and coupled dynamics: distributed approaches. International Journal of Systems Science, 2024, 55(9):1924-1946.

The WLT has a central place in the GRB system, stressing the need of auditing and monitoring at every phase, from project design and development to construction and settlement. The thoroughness and real-time nature of GRB may be guaranteed by developing a system based on the whole lifespan.

2.It is recommended to elaborate more on the potential impact of these estimation methods on the model's predictions in the discussion section and suggest ways to improve data collection and processing in future research. Future studies could consider incorporating data from more actual projects to validate the model's predictive capabilities, especially when applied to different regions and types of building projects.

Reply: Thank you for your constructive feedback on the manuscript. Under your guidance, the potential impact of the estimation method on model predictions and future research have been revised as follows:

Although this study has achieved some success, there are still some limitations, such as the following ways that affect the model's predictions. The estimation of model parameters such as pollutant reduction rate and greening rate is based on local data from example projects. Prediction bias may result from variations in technology, size, or geographical circumstances in real-world projects, such as increased energy use for insulation repair in cold climates. Using expert surveys to get precise values for restricting variables may result in incomplete data. Future research may incorporate actual project data from different climate zones and building types to confirm the applicability of the model in many situations. Real-time data collection of construction energy consumption, garbage disposal, and other indicators, when combined with BIM and Internet of Things sensors, can replace some manual statistical indicators. To capture short-term fluctuations, an adaptive time step is also added to the state variable calculation.

3.Although the conclusion section summarizes the main points, it does not delve deeply into future research directions and practical application challenges of the method. It is suggested that the authors provide more analysis on potential practical issues and propose corresponding solutions, making the conclusions more forward-looking and practically guiding.

Reply: Thank you very much for your valuable feedback on the manuscript. As the discussion section has already covered the content of the conclusion section, which is too redundant, it has been deleted. Potential practical issues and solutions have been added to the discussion section. The specific modifications are as follows.

Although this study has achieved some success, there are still some limitations, such as the following ways that affect the model's predictions. The estimation of model parameters such as pollutant reduction rate and greening rate is based on local data from example projects. Prediction bias may result from variations in technology, size, or geographical circumstances in real-world projects, such as increased energy use for insulation repair in cold climates. Using expert surveys to get precise values for restricting variables may result in incomplete data. Future research may incorporate actual project data from different climate zones and building types to confirm the applicability of the model in many situations. Real-time data collection of construction energy consumption, garbage disposal, and other indicators, when combined with BIM and Internet of Things sensors, can replace some manual statistical indicators. To capture short-term fluctuations, an adaptive time step is also added to the state variable calculation.

4.It is suggested to further supplement relevant references.

Reply: Thank you for your constructive feedback on the manuscript. Under your guidance, the relevant references have been added as follows.

7.Danlei Z,Yong H. The Roles and Synergies of Actors in the Green Building Transition: Lessons from Singapore.Sustainability, 2022, 14(20):13264-13264.

8.Sanchez-Squella A, Yanine F, Barrueto A, Parejo. Green energy generation in buildings: Grid-tied distributed generation systems (DGS) with energy storage applications to sustain the smart grid transformation. Journal ofInformation Technology Management, 2020, 12(2):153-162.

5.The format of the references is inconsistent.

Reply: Thank you for your specific revision suggestions on the manuscript. Under your guidance, the reference format has been standardized as follows.

1.Youcef B, Ambrose D, Truong N, Katarina RG. Comprehensive renovation of a multi-apartment building in Sweden: techno-economic analysis with respect to different economic scenarios. Building Research & Information, 2024,5 2(4):463-478.

2.Amin AM, Mia AA M, Bala T. Green finance continuance behavior: the role of satisfaction, social supports, environmental consciousness, green bank marketing initiatives and psychological reactance.Management of Environmental Quality, 2023, 34(5):1269-1294.

3.Kwame ES, Julian J S. Incentive mechanism for promoting the uptake of green building in South Africa. Open House International, 2024, 49 (2): 340-357.

4.Issa Y AA. Lessons learned, barriers, and improvement factors for mega building construction projects in developing countries: review study. Sustainability, 2021, 13(19):10678-10679

6.Please confirm whether there are redundant and unnecessary icons in Figure 3.Meanwhile, there are a few layout issues in Figure 3, such as insufficient border height.

Reply: Thank you very much for your detailed revision suggestions on the manuscript. The redundant and unnecessary icons in Figure 3 have been removed, and the border height has been modified as required, as follows:

Fig. 3 Identification and analysis framework of constraints for GRB

Reviewer #2: This manuscript explores constraints on green renovation of buildings (GRB) using a System Dynamics Model (SDM) based on Whole Lifecycle Theory (WLT) and Green Development Theory (GDT). The topic is timely and relevant, and the proposed framework offers insights useful for advancing sustainable urban development.
Reply: Thank you for your recognition of the manuscript.

- Integrating WLT and GDT within an SDM framework is innovative and provides a clear theoretical foundation.

Reply: Thank you for your evaluation of the manuscript.

- The analysis effectively considers multiple phases of the renovation process, highlighting key factors that influence sustainability and environmental performance.

Reply: Thank you very much for your evaluation of the manuscript.

- The simulation results are informative and adequately validated with real-world economic and demographic data, enhancing the robustness and relevance of the findings.

Reply: Thank you very much for your recognition of the manuscript.

Suggested areas for improvement include:
- Some parts of the manuscript could benefit from improved clarity and flow. A careful revision focusing on concise and straightforward sentence structure would enhance overall readability and accessibility.

Reply: Thank you for your suggestions on the manuscript. The sentence structure and clarity and fluency of the manuscript content have been modified as required. The specific modifications can be found in the entire text.

- Clearer explanations of the variables and parameters used in equations would make the modeling approach easier to follow. Including a summary table clearly defining these variables would be helpful for readers.

Reply: Thank you for your suggestions on the manuscript. Under your guidance, the variable summary table of the modeling method has been added as required, as follows:

List of variables:

: Fuzzy number standardization function judged by researchers

: Fuzzy number standardization function judged by researchers

: Fuzzy number standardization function judged by researchers

: Standardized numerical functions

: Standardized numerical functions

$P$: Factor set

$n$: Researcher code

$ij$: Factor coefficient

$\max$: Maximum value

$\min$: Minimum value

: Overall standardized clear value

: Clear values corresponding to influencing factors

$k$: Standardization Matrix

$C$: Clear Matrix

$Q$: Planning Matrix

$G_{\text{design }}$: Green level indicators during the planning and design phase

$I_{\text{int }}$: Value of indicator 1

$I_{\text{energy }}$: Degree of energy-saving renovation in buildings

$I_{\text{fire }}$: Integrity of fire protection facilities

$I_{\text{passive }}$: Degree of passive measures transformation

$I_{\text{manage }}$: Green operation and maintenance management level

$Q_{\text{life }}$: Evaluation value of residents' quality of life during the usage phase

$S_{\text{safety }}$: Level of community safety intelligence

$S_{\text{water }}$: Water resource collection and recycling level

$S_{\text{comfort }}$: Level of living comfort

$\rho$: Weight coefficient

$\sigma$: Weight coefficient

$\tau$: Weight coefficient

$w$: Weight coefficient

- Enhancing figures with more detailed labels and descriptive captions would assist readers in interpreting the results. Additionally, clearer subheadings within the methodology and results sections could guide readers through the modeling process more effectively.

Reply: Thank you very much for your specific modification suggestions on the manuscript. The subheadings of the chart labels and explanations, as well as the methods and results section, have been modified according to the requirements, as follows:

As shown in **Fig. 3**, the recognition principle system includes the current principles of comprehensiveness, scientificity, directionality, prominence, and operability. The system involves five core dimensions: system, management, technology, environment, and willingness. It breaks through the traditional "technology economy" binary framework and ultimately outputs a structured model of constraint factors, factor interpretation, and correlation analysis.

Fig.6 Dynamic changes in garbage collection volume during different stages of green building renovation

2.1.1 Green Construction Management Organization System Based on the Whole Life Cycle Theory

2.1.2 Analysis of Constraints on Green Building Renovation

- Expanding the discussion of limitations to consider the role of assumptions, data estimation methods, and the potential generalizability of the model to different contexts could help clarify the scope and applicability of the findings.

Reply: Thank you for your valuable feedback on the manuscript. Under your guidance, the discussion on limitations has been expanded as follows.

Although this study has achieved some success, there are still some limitations, such as the following ways that affect the model's predictions. The estimation of model parameters such as pollutant reduction rate and greening rate is based on local data from example projects. Prediction bias may result from variations in technology, size, or geographical circumstances in real-world projects, such as increased energy use for insulation repair in cold climates. Using expert surveys to get precise values for restricting variables may result in incomplete data. Future research may incorporate actual project data from different climate zones and building types to confirm the applicability of the model in many situations. Real-time data collection of construction energy consumption, garbage disposal, and other indicators, when combined with BIM and Internet of Things sensors, can replace some manual statistical indicators. To capture short-term fluctuations, an adaptive time step is also added to the state variable calculation.

- It would be beneficial to explicitly discuss how the findings could inform practical decisions, such as planning, policy-making, investment choices, or stakeholder coordination in green renovation efforts.

Reply: Thank you very much for your detailed revision suggestions on the manuscript. The content related to the research results providing information for practical decision-making has been revised as required, as follows.

Although this study has achieved some success, there are still some limitations, such as the following ways that affect the model's predictions. The estimation of model parameters such as pollutant reduction rate and greening rate is based on local data from example projects. Prediction bias may result from variations in technology, size, or geographical circumstances in real-world projects, such as increased energy use for insulation repair in cold climates. Using expert surveys to get precise values for restricting variables may result in incomplete data. Future research may incorporate actual project data from different climate zones and building types to confirm the applicability of the model in many situations. Real-time data collection of construction energy consumption, garbage disposal, and other indicators, when combined with BIM and Internet of Things sensors, can replace some manual statistical indicators. To capture short-term fluctuations, an adaptive time step is also added to the state variable calculation.

Overall, this manuscript addresses a meaningful issue and presents a sound modeling approach. With careful attention to language clarity, detailed explanation of variables, improved figure presentation, and enhanced practical implications, this work can contribute to research on sustainable building renovation.

Reply: Thank you very much for your suggestions on the manuscript. The clarity of the language, detailed explanations of variables, improvement of charts, and enhancement of practical application significance have all been modified as required. The specific modifications can be found in the full text.
