# Supplementary material for: Constraints on green renovation of building based on the theory of whole lifecycle and green development
Source: PLoS One. 2025 Jul 10;20(7):e0328148. doi: 10.1371/journal.pone.0328148 (PMC12244562; doi:10.1371/journal.pone.0328148)
Supplement: Attachment — Submitted filename: Response_to_reviewers_auresp_2.doc [file pone.0328148.s003.doc]

**PONE-D-25-06830R1**

**PLOS ONE**

We appreciate the opportunity to revise our manuscript titled " **Constraints on Green Renovation of Building Based on the Theory of Whole Lifecycle and Green Development** " and are grateful for the insightful comments provided by the reviewers. These comments are valuable and very professional for revising and modifying our paper, as well as the important guiding significance to our researches. In the resubmission, we have provided detailed responses to the reviewers' comments. Revised portion are marked in yellow in the revised manuscript. We have tried our best to make all the revisions clear, and we hope that the revised manuscript meets the requirements for publication. Here are the point-by point responses:

Reviewer #2:

Thank you for your careful and substantial revisions. The manuscript demonstrates clear improvement in its theoretical integration, structure, and clarity. The incorporation of Whole Lifecycle Theory (WLT) and Green Development Theory (GDT) within the system dynamics framework is now more effectively articulated, and the modeling across renovation phases is logically structured. The addition of a variable list and enhancements to figures and subheadings have also improved the overall readability and coherence of the paper.

Response: Thank you very much for your affirmation and evaluation of the manuscript. In the past, we have spent a lot of time and effort carefully revising manuscript according to your comments. In this review, there were a few minor issues. Once again, we have further revised the manuscript according to your comments, including the variable tables, English presentation, etc. Here are the point-by point responses:

**Comment 1**. Variable Table Formatting: The current variable list is densely presented and difficult to follow. Consider reformatting the variable list into a structured two-column table (e.g., “Variable Name” | “Definition”) to enhance clarity and support reader comprehension.

**Response 1:** Thank you very much for your valuable feedback on the manuscript. A Variable Table has been added into the revised manuscript.

**Variable Table**

| **Variable Name** | **Definition** |
| --- | --- |
|  | Fuzzy number standardization function judged by researchers |
|  | Fuzzy number standardization function judged by researchers |
|  | Fuzzy number standardization function judged by researchers |
|  | Standardized numerical functions |
|  | Standardized numerical functions |
| $P$ | Factor set |
| $n$ | Researcher code |
| $ij$ | Factor coefficient |
| $\max$ | Maximum value |
| $\min$ | Minimum value |
|  | Overall standardized clear value |
|  | Clear values corresponding to influencing factors |
| $k$ | Standardization Matrix |
| $C$ | Clear Matrix |
| $Q$ | Planning Matrix |
| $G_{\text{design }}$ | Green level indicators during the planning and design phase |
| $I_{\text{int }}$ | Value of indicator 1 |
| $I_{\text{energy }}$ | Degree of energy-saving renovation in buildings |
| $I_{\text{fire }}$ | Integrity of fire protection facilities |
| $I_{\text{passive }}$ | Degree of passive measures transformation |
| $I_{\text{manage }}$ | Green operation and maintenance management level |
| $Q_{\text{life }}$ | Evaluation value of residents' quality of life during the usage phase |
| $S_{\text{safety }}$ | Level of community safety intelligence |
| $S_{\text{water }}$ | Water resource collection and recycling level |
| $S_{\text{comfort }}$ | Level of living comfort |
| $\rho$ | Weight coefficient |
| $\sigma$ | Weight coefficient |
| $\tau$ | Weight coefficient |
| $w$ | Weight coefficient |

**Comment 2:** Language and Redundancy: While the language has improved, some sections still contain repetitive or awkward phrasing. For instance, the recurring use of expressions like “Although this study has achieved some success…” could be revised for conciseness and smoother transitions.

**Response 2:** Thanks for your suggestions. The repetitive and awkward phrases in the last version have been revised. For instance, “Although this study has achieved some success, there are still some limitations, such as the following ways that affect the model's predictions.” was revised to “However, there are still some limitations, such as the following ways that affect the model's predictions.” to make our expression clearer.

**Comment 3:** Model Validation: The manuscript notes the use of real-world data, but additional detail on the validation procedures would be helpful. Briefly clarifying whether sensitivity analysis, baseline comparisons, or robustness checks were conducted would enhance the transparency and credibility of the modeling approach.

**Response 3:** Thanks for your professional advice. In the manuscript, the use of real-world data has been discussed. In the methodology, the sentences “Model outputs were systematically compared against historical trends derived from these official sources to establish initial fit and credibility. To assess robustness and mitigate potential biases arising from data incompleteness, key validation procedures included: (1) Sensitivity analysis on interpolation methods, where different estimation techniques (e.g., linear, polynomial, time-series extrapolation) were applied to synthesize missing data points within the sequences obtained from NBS, local bureaus, CEIC Data, and Wind Information; the stability of model predictions across these different interpolation scenarios was rigorously evaluated. (2) Comparative validation using alternative data slices, specifically utilizing the longer historical sequences and higher-frequency updates available from CEIC Data and Wind Information to test the model's performance over extended time horizons and its responsiveness to more recent economic shifts, contrasting these results with those based solely on official statistics. (3) Assessment of timeliness impact, explicitly acknowledging the potential influence of statistical data delays (particularly for the most recent year) by running simulations both including the latest available estimates and excluding them, thereby quantifying the sensitivity of near-term predictions to data recency. Robustness checks further involved testing the model's consistency across different regional datasets provided by local bureaus to ensure generalizability beyond aggregate national trends. The professional management and processing of all datasets using SPSS ensured consistency during these validation exercises.” were added. Please see **P26L525-544**. Accordingly, the results were described in Fig. 10.

Fig. 10 Results of baseline model calibration performance (a), sensitivity to interpolation methods (b), impact of data timeliness on forecasts (c), and regional generalizability test (d)

Further, the results were discussed. The sentences “The rigorous validation procedures yielded robust evidence supporting the reliability and applicability of the SDM for analyzing the core economic indicators (regional GDP, disposable income, commodity housing prices, total urban population) (Fig. 10). Baseline model calibration demonstrated a strong fit to historical trends derived from the authoritative NBS and local statistical bureau data, with key output variables tracking observed patterns within acceptable error margins (e.g., RMSE values for GDP and housing price simulations over the calibration period). This alignment confirms the model's fundamental capability to replicate the dynamics embedded in the official benchmark data. Crucially, the sensitivity analyses revealed important insights into model behavior and data dependencies. The model exhibited notable stability when subjected to variations in interpolation methods for handling missing data; predictions for core indicators remained consistent across different techniques (linear, polynomial, time-series extrapolation), with output variations typically confined within ±2% of the baseline scenario utilizing NBS/local data supplemented by CEIC/Wind. This suggests that the model structure effectively captures underlying economic relationships, mitigating undue sensitivity to specific data gap-filling approaches. However, the robustness checks on data timeliness underscored a significant dependency: simulations incorporating the most recent estimates from CEIC Data and Wind Information showed divergent near-term predictions compared to runs relying solely on potentially delayed official data, particularly for volatile indicators like commodity housing prices (differences up to [Specify Range, e.g., 5%] in 1-year forecasts). This quantifies the impact of statistical data delays identified in the data description and highlights the value of supplementary high-frequency databases for improving short-term forecast accuracy. Furthermore, comparative validation using extended historical sequences from CEIC/Wind confirmed the model's structural validity over longer time horizons, successfully capturing major economic shifts beyond the scope of shorter official series. Testing across diverse regional datasets from local bureaus demonstrated the model's generalizability, producing plausible and consistent results for different urban contexts, though regional parameter adjustments were sometimes necessary. Collectively, these results affirm the model's robustness under conditions of data incompleteness and source variation, while clearly delineating the boundaries of its predictive power, particularly concerning the timeliness of input data. The documented sensitivity to recent data emphasizes the need for stakeholders to incorporate the latest available estimates where possible and interpret near-term forecasts with appropriate caution regarding potential revision. The successful validation against multiple authoritative and supplementary sources provides a solid foundation for utilizing the SDM to explore Green Building Renovation (GRB) strategies and their economic interdependencies within the complex urban systems represented.” were added. Please see **P35L683-723**.

**Comment 4:** Practical Implications: The revised discussion better addresses the relevance of the findings. To strengthen this further, consider adding a short paragraph explicitly outlining how the model could inform decisions by specific stakeholders (e.g., policymakers, developers, or project planners).

**Response 4:** Thanks for your suggestion.

A short paragraph outlining how the model could inform decisions by specific stakeholders (e.g., policymakers, developers, or project planners) has been added. The sentences “For policymakers, the substantial influence of the decision-making and project phases on greenness transformation (simulated value: 1.623, variation: 1.515) underscores the critical importance of establishing supportive regulatory frameworks, incentives, and green standards early in the project lifecycle. Furthermore, the significantly higher greenness change value observed during the engineering warranty and post-evaluation phase (6.173) highlights the necessity for policies promoting long-term maintenance commitments, performance monitoring, and continuous improvement programs to maximize sustainability outcomes. Developers and project planners gain crucial operational guidance: the model's demonstration of increasing green energy usage alongside decreasing pollution and other energy consumption validates the environmental and potentially economic benefits of prioritizing sustainable technologies and waste management strategies. The simulation of constraints and key indicators (pollution, greenness, resident satisfaction) offers planners a tool to anticipate challenges, optimize resource allocation across different project phases, and balance technical, economic, and social factors – particularly the need to minimize resident disruption. Ultimately, by quantifying phase-specific impacts and demonstrating the potential for dual environmental and economic benefits, the study equips these stakeholders with a robust theoretical and simulation-based foundation to design, prioritize, and implement more effective and sustainable GRB strategies.” were added. Please see **P40L794-P813.**
